# Don’t Look Back and Spray! Risk of Endoscope Adhesion with Hemospray Application in Retroflexion in a Pediatric Patient

**DOI:** 10.1097/PG9.0000000000000336

**Published:** 2023-07-17

**Authors:** Sarah T. Edwards, Diana G. Lerner, Moises Alatorre Jimenez, Thomas M. Attard

**Affiliations:** From the * Division of Gastroenterology, Children’s Mercy Kansas City, Kansas City, MO; † Department of Pediatrics, School of Medicine, University of Missouri Kansas City, Kansas City, MO; ‡ The Department of Pediatrics, Section of Gastroenterology, Hepatology, and Nutrition, Medical College of Wisconsin, Milwaukee, WI.

**Keywords:** complications, endoscopy, gastrointestinal bleeding, hemostatic powder

## Abstract

Although the vast majority of recognized pediatric upper gastrointestinal bleeding (GIB) resolves spontaneously, gastrointestinal hemorrhage is the most common indication for urgent or emergent therapeutic endoscopy in pediatric practice. The application of hemostatic powders, including TC-325 (Hemospray, Cook Medical, Winston-Salem, NC, USA), has shown considerable impact on the control of acute bleeding, with the advantage of potentially covering an extensive area and requiring less technical expertise. We report a case of transient adherence of an esophagogastroduodenoscopy following Hemospray application in a 22-month-old with upper GIB. Our experience does not detract from the significant gains in the management of pediatric GIB from Hemospray; however, it does raise a cautionary note toward the application technique utilized.

## INTRODUCTION

Although the vast majority of recognized pediatric upper gastrointestinal bleeding (GIB) resolves spontaneously (1), gastrointestinal hemorrhage is the most common indication for urgent or emergent therapeutic endoscopy in pediatric practice (2). The therapeutic strategy and outcome in GIB are determined by the acuity and severity of bleeding, patient frailty along with the presumed source of bleeding. When a patient requires therapeutic endoscopy, it has historically been recommended to employ at least 2 different modalities for an increased likelihood of successful and lasting hemostasis. There is a variable spectrum of expertise to perform endoscopic interventions, which can impact outcomes (3).

The application of hemostatic powders, including TC-325 (Hemospray, Cook Medical, Winston-Salem, NC, USA), has shown considerable impact on the control of acute bleeding, with the advantage of potentially covering an extensive area and requiring less technical expertise. Hemospray activates upon contact with moisture and becomes cohesive and adhesive, forming a stable mechanical barrier and sealing the bleeding site. It is neither metabolized nor absorbed, minimizing the risk of systemic toxicity.

Hemospray is established as an adjunct or salvage treatment in GIB. In adults, short-term success in hemostasis is high (>90%) but rebleeding within 30 days is reported in a subgroup of patients (10–30%). Although one application of 20 grams (single canister container) of Hemospray is usually needed, repeated application is reported as both safe and effective. Effective as monotherapy in the treatment of pediatric, nonvariceal upper gastrointestinal bleeding, it was also shown to be comparable to other conventional endohemostatic techniques with a similar initial hemostatic and subsequent rebleeding rate (4).

Adverse events related to Hemospray have been noted but are rare. There are reports of transient adherence of the endoscope to the esophageal mucosa after use in retroflexion (5–7). The proposed mechanism is adherence from layered inactive bentonite on the surface of the endoscope that is activated once the straightened endoscope is withdrawn and in contact with moist esophageal mucosa. Although most cases reported were resolved with irrigation or manipulation (pushing, twisting, or pulling) without incurring harm to the patient, in one instance the endoscope was retained with removal at a later time. Despite the established use of Hemospray in children, there have not been reports of scope retention. Herein we report transient, adherence of an esophagogastroduodenoscopy following Hemospray application in a 22-month-old with upper GIB.

## CASE REPORT

**FIGURE 1.**
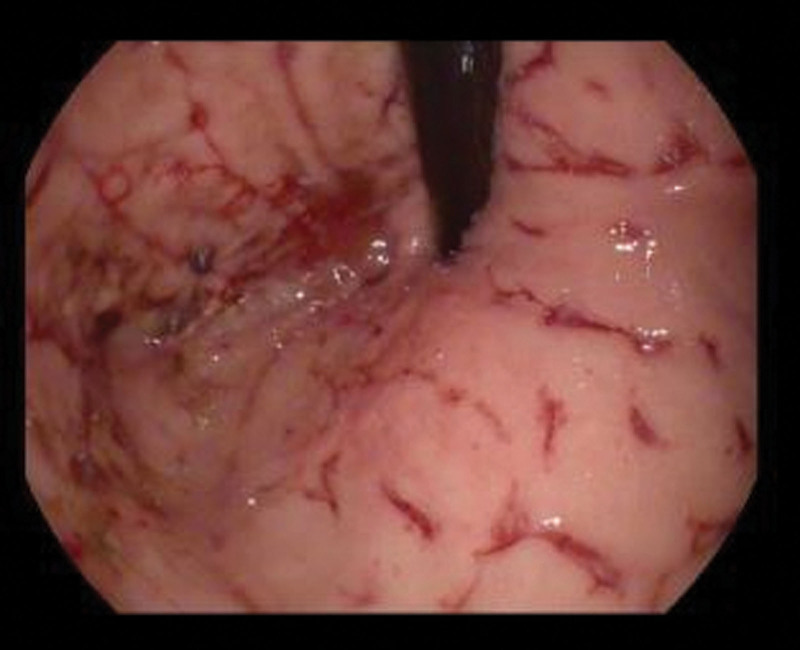
Gastric cardia with deep, serpentine ulcerations with slow, diffuse active oozing of blood.

**FIGURE 2.**
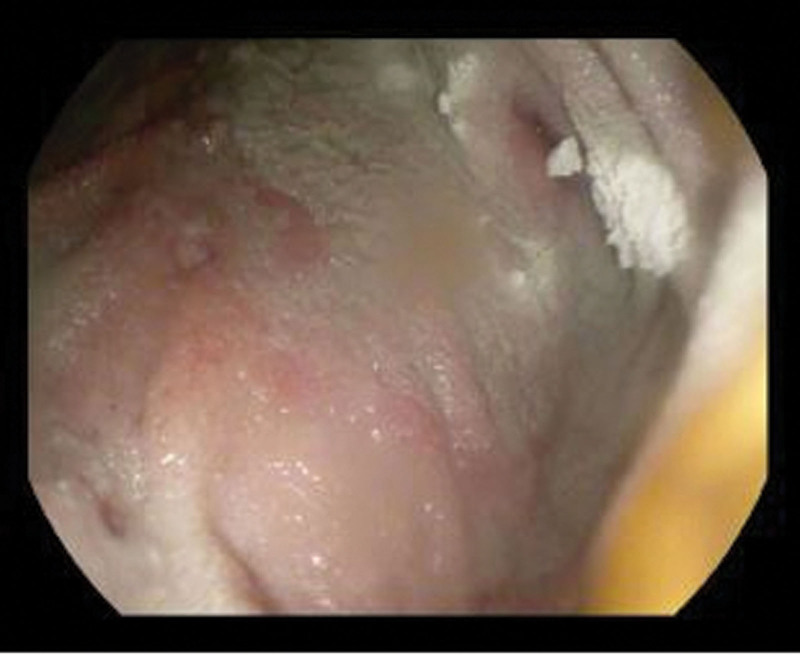
Gastric mucosa status post-treatment with Hemospray.

Our patient, a 22-month-old previously healthy male, presented to our facility with a 2-day history of fatigue and anorexia and a 1-day history of low-grade fever and the passage of black tarry stools. In the emergency department, he had 1 episode of large hematemesis. He was found to be anemic (hemoglobin 8.2 gm/dL), leukocytopenic (WBC count 2.7 × 10^3^/mcL), and hyponatremic (127 mmol/L) acidotic (carbon dioxide 16 mmol/L), with significant transaminasemia (aspartate aminotransferase 553 unit/L and alanine aminotransferase 417 unit/L). Coagulation studies were normal. Chest, abdominal x-ray, and abdominal ultrasound were unremarkable. At admission, his weight was 11.3 kg (z-score −0.21). He received 1 unit of packed red blood cells with an increase in hemoglobin to 10.4 gm/dL. He was started on a continuous infusion of octreotide and an intravenous proton pump inhibitor (PPI). The hemoglobin progressively decreased to 6.7 gm/dL 8 hours after transfusion. Consequently, the patient was taken to the operating room for an emergent endoscopy. The patient was intubated, and an EG-2790i endoscope was used for the procedure. Endoscopy showed deep, serpentine ulcerations with slow, diffuse, active oozing of blood throughout the gastric cardia and gastric body. The visualization of the most severe ulcerations was seen in retroflexion (Fig. 1). Hemospray was applied diffusely to the gastric cardia and gastric body in retroflexion, covering both the mucosa and the endoscope (Fig. 2). Upon removal, there was significant resistance when the distal half of the endoscope that was covered in Hemospray reached the esophagus. An increase in traction with withdrawal was needed to release it from the esophagus. No significant esophageal trauma was noted upon reintubation. The patient was taken to the pediatric intensive care unit for close monitoring, continued octreotide, and PPI infusion. Adenovirus polymerase chain reaction was positive. He maintained his hemoglobin postoperatively and was able to be weaned off the octreotide. Transaminases were trending down at discharge, and at 1-month follow-up had normalized. Repeat endoscopy 3 months later was normal.

## DISCUSSION

The management of significant GIB in pediatric patients can be a medical-surgical emergency. There is significant mortality and morbidity from bleeding, which is determined in large part by patient frailty and coexisting chronic illnesses (8). The use of hemostatic powder has revolutionized the management of GI bleeding, considering its effectiveness and the relatively low skill level needed for its application.

Our experience does not detract from the significant gains in management of pediatric GIB from Hemospray; however, it does raise a cautionary note toward the application technique utilized. The layering of inactivated powder on activated powder, adhering to the endoscope results in a potentially adhesive effect between the endoscope and esophageal mucosa once the straightened endoscope is withdrawn. Countermeasures can include limiting the use of hemostatic powder when in retroflexion, if possible, moistening the endoscope surface if covered by (paler colored) hemostatic powder, and, possibly aggressive lavage if adherence happens, although this is unproven as a potential reversal technique.

Our patient suffered no immediate or long-term adverse effects and recovered from the procedure uneventfully. Our impression is that the degree of traction that needed to be applied to extract the endoscope was excessive, and we were concerned that it might be potentially hazardous. Other maneuvers, such as the use of a second endoscope next to the first or leaving the endoscope in situ, were not an option in our patient due to patient size and concerns for the ability to monitor for rebleeding, respectively. Avoidance of an excessive amount of hemostatic powder in bleeding lesions at the gastroesophageal junction has been recommended unless the endoscopist is able to place the endoscope proximal to the lesion. In cases where extracting the endoscope is not possible, it has been suggested to leave the scope in situ for 48–72 hours, since the hemostatic powder will completely be eliminated in the GI tract by 48–72 hours. Ultimately, in our case, constant traction for several seconds with gentle side-to-side rotation extricated the endoscope, which was then withdrawn (7).

In summary, as in any other therapeutic modality, the use of hemostatic powder should be limited to the amount needed to achieve hemostasis. Additional caution needs to be exerted if applying in retroflexion, for cardia lesions as in our case. A paler colored layering of Hemospray on the endoscope may signal the presence of inactivated powder with the potential to cause adhesion, and this could be countered by careful irrigation.

## ACKNOWLEDGMENTS

Informed consent was obtained from the patient’s parent for the publication of the case details.
